# Evaluating perceived obstetric anesthesia training needs in residency education: a Taiwanese national survey

**DOI:** 10.1080/10872981.2026.2731792

**Published:** 2026-09-15

**Authors:** Li-Min Hu, Chia-Chih Liao, Feng-Cheng Chang, Wei-Hsiang Chao, Wen-Shan Cheng, Chung-Hsien Chaou

**Affiliations:** a Department of Anesthesiology, Linkou Chang Gung Memorial Hospital, Taoyuan, Taiwan; b Division of Medical Education, Graduate Institute of Clinical Medical Science, College of Medicine, Chang Gung University, Taoyuan, Taiwan; c Taiwan Society of Anesthesiologists, Taipei, Taiwan; d Department of Emergency Medicine, Linkou Chang Gung Memorial Hospital, Taoyuan, Taiwan; e Medical Education Research Center, Chang Gung Memorial Hospital, Chang Gung University, Taoyuan, Taiwan

**Keywords:** Declining birthrate, medical education, needs assessment, obstetric anesthesia, residency training

## Abstract

**Background:**

Taiwan’s declining birthrate has reduced obstetric case volume and may threaten obstetric anesthesia training. We examined training experiences, self-perceived competencies, and educational needs in Taiwan to inform feasible curricular strategies.

**Methods:**

We conducted a cross-sectional nationwide anonymous online survey of anesthesiology residents and attending anesthesiologists in Taiwan (October 1–December 31, 2024). The 37-item questionnaire covered demographics, self-assessed competencies and confidence, satisfaction with current training, and future training preferences, with open-ended items on barriers to additional training. Descriptive statistics, independent-samples t tests, one-way ANOVA, Pearson correlations, and linear regression were performed.

**Results:**

A total of 145 valid responses were analyzed (112 attendings, 33 residents), yielding an overall response rate of 8.3% (attendings 7.8%, residents 10.4%). Self-assessed competence increased with greater clinical seniority and longer obstetric anesthesia training duration. Highest-rated competencies were neuraxial anesthesia for cesarean delivery (mean 7.93) and airway management during general anesthesia in obstetric patients (7.82), whereas confidence was lower for managing cardiac parturients (6.21) and neonatal resuscitation (5.89). Attendings reported higher overall confidence in independently performing obstetric anesthesia than residents (8.00 vs 6.52, *p *< 0.001). Male respondents were significantly older than female respondents (mean 41.9 vs. 35.4 years, *p *< 0.001). Correspondingly, male respondents reported higher confidence than female respondents for independent practice (8.05 vs 7.08, *p *< 0.001) and for managing cardiac parturients (6.63 vs 5.59, *p *= 0.001). In multivariable regression, confidence in managing cardiac parturients (*β *= 0.61, 95% CI 0.217–0.997; *p *= 0.002) and competence in neuraxial anesthesia for cesarean delivery (*β *= 1.214, 95% CI 0.190–2.238; *p *= 0.020) independently predicted overall confidence.

**Conclusions:**

Obstetric anesthesia competence and confidence were associated with training exposure, yet gaps persisted in high-risk and emergency domains and differed by gender. Programs should prioritize simulation-based training for rare critical events and develop inter-institutional collaborations to strengthen competency acquisition amid declining obstetric volumes.

## Introduction

As birth rates across Europe, Asia, and the Americas reach record-low levels, more than 50% of the global population currently resides in nations where total fertility rates have fallen below the replacement threshold—a demographic shift that leads to population aging, a shrinking labour force, and increased healthcare expenditures, placing growing pressure on the healthcare system [1–4]. In 2024, Taiwan’s population stood at approximately 23,400,220, yet recorded a historical low of only 134,856 births—a persistent decline in fertility that undermines both healthcare operations and clinical training quality, with these challenges being particularly pronounced in the field of obstetric anaesthesia [5–8]. Taiwan’s maternal mortality rate has shown an overall upward trend over the past two decades, reaching 18 per 100,000 live births in 2022—approximately four times higher than Japan’s and 1.5 times higher than South Korea’s [9,10]. This rise may be associated with demographic and obstetric changes, particularly delayed childbearing and the increasing prevalence of caesarean deliveries [11,12]. Obstetric anaesthesia is a demanding subspecialty where the core of education lies in building a strong foundation in theory and practice—encompassing pregnancy-related physiology and pharmacology, neuraxial anaesthesia, intraoperative monitoring, and the management of life-threatening conditions such as postpartum haemorrhage and preeclampsia—while requiring effective interdisciplinary communication and collaboration with obstetricians as essential components of clinical practice [13–15]. Anaesthesiology residency training in Taiwan spans 48 months, integrating core clinical education with subspecialty rotations to ensure the development of comprehensive competence and sound clinical decision-making skills. The curriculum includes a six-month elective period, allowing residents to tailor their training toward specific interests or career plans, with opportunities to pursue advanced subspecialty rotations—such as cardiac, paediatric, obstetric, pain, or critical care anaesthesia—after completing core requirements. Yet, within this structured framework, the 2024 curriculum revision reduced the mandatory obstetric anaesthesia rotation from five months (60 cases) to just three months (40 cases). Given these reduced thresholds, obstetric exposure alone is unlikely to provide sufficient technical proficiency, especially when traditional rotation-based models struggle to capture rare but critical emergencies in a low-volume environment [16]. This concern is further underscored by established empirical benchmarks, which suggest that residents require roughly 45 spinal and 60 epidural attempts to achieve a 90% success rate; consequently, as declining case volumes increasingly limit training opportunities, the resulting challenges to technical proficiency and emergency response capabilities not only impede professional development but also potentially compromise maternal anaesthesia safety and the overall quality of obstetric care [17–20].

Therefore, systematic evaluation of current training gaps is essential to ensure patient safety and professional development. To address this, we conducted a nationwide survey of anaesthesiology attendings and residents in Taiwan to examine the relationship between training duration, clinical confidence, and unmet educational needs.

## Materials and methods

### Study design

This was a cross-sectional national survey study. The survey was administered via SurveyCake (version 5.18; 25sprout, LLC., Taiwan), an online platform used for anonymous data collection. The participants were informed through the survey invitation that this study aimed to evaluate the current status and training needs of obstetric anaesthesia education in anaesthesiology residency programmes in Taiwan.

The invitation explained the background and purpose of the research, indicated that participation was voluntary, and that responses would be collected anonymously. Only anaesthesiology residents and attending anesthesiologists were invited to participate. This study was approved by the Institutional Review Board of Chang Gung Medical Foundation Institutional Review Board (IRB No. 202401358B0). This study was conducted in accordance with the principles of the Declaration of Helsinki. Participation was voluntary and anonymous. Completion of the online questionnaire was considered as implied informed consent. As no identifiable personal information was collected and the survey posed minimal risk, the Institutional Review Board approved a waiver of written informed consent.

### Questionnaire development and dissemination

The questionnaire development was informed by a published set of obstetric anaesthesia core competencies for US residency programmes, including domains such as neuraxial and general anaesthesia for labour and caesarean delivery, management of obstetric crises (e.g., postpartum haemorrhage, eclampsia, high spinal block), maternal physiology and pharmacology [21]. The questionnaire was subsequently reviewed and refined through three rounds of expert consultation to evaluate content relevance, clarity, and applicability to the Taiwanese training context. The expert panel included an obstetric anesthesiologist with over 20 years of clinical experience and a biostatistics specialist. The preliminary questionnaire was then pilot-tested with three anaesthesia trainees of different residency years, and minor revisions were made to improve wording, survey flow, and usability. The final 37-item questionnaire covered four domains: (1) demographic characteristics, (2) self-assessed skills and confidence in obstetric anaesthesia, (3) satisfaction with current training, and (4) perceived needs and preferences for future training models, with additional open-ended questions regarding further training. The questionnaire was designed as a needs-assessment instrument based on self-reported perceptions and was not intended to provide an objective assessment or certification of clinical competence.

The questionnaire was distributed via email to all board-certified anesthesiologists and current anaesthesiology residents in Taiwan. The email invitation contained an embedded link to the electronic survey and was distributed nationwide to all board-certified anesthesiologists (*n *= 1,431) and anaesthesiology residents currently in training (*n *= 316). The survey was open from October 1, 2024, to December 31, 2024. One reminder email was sent to participants on December 1, 2024 during the survey period to encourage completion.

### Data analysis

All statistical analyses were performed using IBM SPSS Statistics for Windows, Version 24.0. Descriptive statistics, including means, standard deviations, and percentages, were used to summarise participants’ demographic characteristics such as age, gender, geographic region, hospital level, and duration of obstetric anaesthesia training. Pearson correlation analysis was conducted to assess the internal consistency of self-assessed competencies and to explore the integration between knowledge and practical skills. To examine differences in self-assessed clinical competence, confidence, and training satisfaction across groups, independent-samples t-tests were used for binary variables (e.g., gender), and one-way analysis of variance (ANOVA) was applied to compare multiple groups (e.g., training duration, years of experience). To identify factors associated with the confidence to independently perform obstetric anaesthesia, linear regression analyses were conducted. Univariate regression was used to identify significant predictors, followed by multivariate regression to determine independent predictors after adjusting for potential confounders. Regression results are reported as *β* coefficients, *p*-values, and 95% confidence intervals (95% CI).

Qualitative responses regarding barriers to voluntary training were analysed following Braun and Clarke’s six-phase thematic analysis framework [22]. To ensure rigour, two researchers (anesthesiologists and medical educators) independently coded the data and resolved discrepancies through iterative discussion until consensus was reached. The analysts practiced reflexivity throughout the process, critically examining their clinical assumptions and educational roles to ensure that the interpretation accurately reflected participants' perspectives rather than pre-existing professional biases.

## Results

### Participant characteristics

A total of 145 valid responses were collected, including responses from 112 attending anesthesiologists and 33 residents. The overall response rate was 8.3%, with 7.8% among attending anesthesiologists (112/1431) and 10.4% among residents (33/316). As presented in Table 1, the mean age of respondents was 39.3 years (SD = 10.1). Regarding gender distribution, 86 participants (59.3%) were male and 59 (40.7%) were female. About 3 quarters of the participants (77.2%) were attending physicians, with one third of them (33.8%) having less than five years of practice. The demographic composition of our sample closely mirrors the national distribution of anaesthesiology clinicians in Taiwan. Specifically, the ratio of attending physicians to residents (77.9% vs. 22.1%) and the gender distribution (67.6% male vs. 32.4% female) are highly consistent with the official membership data from the Taiwan Society of Anesthesiologists (82.5% vs. 17.5% and 67.9% vs. 32.1%, respectively).

**Table 1.** Respondent characteristics.

	Count (mean)	Percentage (SD)
Age	39.3	10.1
**Gender**		
Male	86	59.3
Female	59	40.7
**Years of experience**		
R1	3	2.1
R2	7	4.8
R3	9	6.2
R4	14	9.7
≤5 years as attending	49	33.8
>5 years as attending	63	43.4
**Training hospital location**		
Northern region	96	66.2
Central region	19	13.1
Southern region	26	17.9
Eastern region	4	2.8
**Hospital level**		
Medical centre	134	92.4
Regional hospital	11	7.6
**Training duration**		
Untrained	2	1.4
1–3 months	31	21.4
3–5 months	30	20.7
>5 months	82	56.6

Note: R1, R2, R3, and R4 indicate the first, second, third, and fourth years of residency training, respectively. Hospital level was classified according to Taiwan’s national hospital accreditation system, in which medical centres represent the highest accreditation level and regional hospitals the next level.

The respondents were distributed across different areas of Taiwan, with most participants receiving training at medical centres (92.4%). Regarding the duration of obstetric anaesthesia training, over half of the respondents (56.6%) had undergone more than five months of training, while 20.7% received 3–5 months, 21.4% received 1–3 months, and only 1.4% had not yet commenced any training. When asked about their willingness to voluntarily participate in obstetric anaesthesia rotations, 56.6% indicated a positive inclination, whereas 43.4% expressed unwillingness.

### Self-assessment of obstetric anaesthesia competence

As shown in Table 2, respondents reported the highest confidence in neuraxial anaesthesia for caesarean delivery (mean 7.93), airway management during general anaesthesia (7.82), and anaesthetic drug selection (7.61). Pain management skills, including labour and post-caesarean analgesia, also rated highly (7.61 and 7.46). Knowledge of maternal physiology and pharmacology was moderate to high (6.88–7.57). Critical care skills generally ranged from 6 to 7, with lower ratings for amniotic fluid embolism (6.35) and neonatal resuscitation (5.89). Overall confidence in independently performing obstetric anaesthesia was high (7.66), but lower for high-risk patients with cardiovascular disease (6.21). Training satisfaction was moderate (rotation duration 7.05; content 6.93).

**Table 2.** Obstetric anaesthesia competencies: resident–attending comparison.

	Resident		Attending		*p*-value	Overall	
	Mean	(SD)	Mean	(SD)		Mean	(SD)
Age	30.4	2.6	41.9	10.0	<0.001	39.3	10.1
Case volume during one-month rotation	36.7	23.84	42.4	29.4	0.311	41.1	28.2
Familiarity with physiological changes	6.03	1.53	7.61	1.37	<0.001	7.25	1.55
Familiarity with general anaesthetics selection	6.03	1.53	7.61	1.37	<0.001	7.61	1.45
Airway management for general anaesthesia	6.94	1.56	8.08	1.21	<0.001	7.82	1.38
Neuraxial anaesthesia for caesarean delivery	7.30	1.49	8.12	1.21	0.002	7.93	1.32
Familiarity with the effects of local anaesthetics	6.79	1.56	7.80	1.31	<0.001	7.57	1.43
Familiarity with the safety of local anaesthetics	6.61	1.37	7.73	1.36	<0.001	7.48	1.44
Familiarity with enhanced recovery after caesarean delivery protocols	5.42	1.94	6.65	1.90	0.002	6.37	1.97
Postoperative pain management after caesarean delivery	6.76	1.42	7.66	1.40	0.001	7.46	1.45
Management of accidental dural puncture	6.67	1.58	7.76	1.38	<0.001	7.51	1.49
Management of local anaesthetic systemic toxicity	6.12	1.64	7.45	1.50	<0.001	7.14	1.62
Management of high or total spinal block	6.15	1.52	7.62	1.45	<0.001	7.28	1.58
Management of postpartum haemorrhage	6.03	1.74	7.54	1.37	<0.001	7.20	1.59
Management of preeclampsia and eclampsia	5.79	1.83	7.28	1.50	<0.001	6.94	1.70
Management of amniotic fluid embolism	5.03	1.65	6.74	1.61	<0.001	6.35	1.77
Management of maternal resuscitation	5.36	1.58	7.12	1.61	<0.001	6.72	1.76
Management of neonatal resuscitation	4.3	1.81	6.35	1.85	<0.001	5.89	2.02
Confidence in independently performing anaesthesia	6.52	1.64	8.00	1.28	<0.001	7.66	1.50
Confidence in managing cardiac parturients	4.70	1.93	6.65	1.80	<0.001	6.21	2.00
Satisfaction with training duration	6.45	1.48	7.22	2.05	0.047	7.05	1.96
Satisfaction with training content	6.24	1.62	7.13	2.09	0.025	6.93	2.02

### Comparison of residents and attending anesthesiologists

As shown in Table 2, attending anesthesiologists were older than residents (41.9 vs. 30.4 years, *p *< 0.001), with no difference in reported case volume. Attendings rated higher familiarity across most knowledge and technical domains (all *p *< 0.001). They also reported greater confidence in managing obstetric emergencies. Confidence levels were higher among attendings both for independently performing obstetric anaesthesia (8.00 vs. 6.52, *p *< 0.001) and for managing high-risk cardiac parturients (6.65 vs. 4.70, *p *< 0.001). Satisfaction with training duration and content was modestly higher among attendings.

### Gender differences in self-assessed competencies

As shown in Table 3, male respondents were significantly older than female respondents (41.9 vs. 35.4 years, *p *< 0.001), but no gender difference was found in case volume. In self-assessment, male respondents rated their confidence more highly than female respondents across most skill and knowledge domains. Significant gender gaps were also noted in emergency management. Confidence levels were higher among males, both in independently performing obstetric anaesthesia (8.05 vs. 7.08, *p *< 0.001) and in managing high-risk patients with cardiovascular disease (6.63 vs. 5.59, *p *= 0.001).

**Table 3.** Gender-based comparison of characteristics and competencies.

	Male		Female		*p*-value
	Mean	(SD)	Mean	(SD)	
Age	41.9	11.1	35.4	6.90	<0.001
Case volume during one-month rotation	41.6	27.8	40.3	28.2	0.783
Familiarity with physiological changes	7.61	1.47	6.73	1.53	0.001
Familiarity with general anaesthetics selection	7.94	1.22	7.14	1.63	0.001
Airway management for general anaesthesia	8.03	1.27	7.51	1.49	0.025
Neuraxial anaesthesia for caesarean delivery	8.14	1.26	7.63	1.35	0.021
Familiarity with the effects of local anaesthetics	7.74	1.43	7.08	1.37	0.006
Familiarity with the safety of local anaesthetics	7.01	1.75	6.69	1.6	0.279
Familiarity with enhanced recovery after caesarean delivery protocols	6.67	2.07	5.93	1.75	0.027
Postoperative pain management after caesarean delivery	7.8	1.32	6.95	1.49	<0.001
Management of accidental dural puncture	7.82	1.36	7.07	1.57	0.003
Management of local anaesthetic systemic toxicity	7.45	1.54	6.69	1.65	0.005
Management of high or total spinal block	7.55	1.52	6.9	1.62	0.015
Management of postpartum haemorrhage	7.35	1.52	6.98	1.67	0.174
Management of preeclampsia and eclampsia	7.19	1.69	6.58	1.65	0.033
Management of amniotic fluid embolism	6.66	1.73	5.90	1.74	0.005
Management of maternal resuscitation	7.07	1.70	6.20	1.73	0.002
Management of neonatal resuscitation	6.24	2.00	5.37	1.96	0.005
Confidence in independently performing anaesthesia	8.05	1.13	7.08	1.77	<0.001
Confidence in managing cardiac parturients	6.63	1.94	5.59	1.94	0.001

### Predictors of confidence in independently performing obstetric anaesthesia

As shown in Table 4, a linear regression analysis was conducted with ‘confidence in independently performing obstetric anaesthesia’ as the dependent variable. In the univariate analysis, several variables were significantly and positively associated with confidence, including ‘familiarity with maternal physiological changes’ (*β *= 1.51, *p *< 0.001), ‘neonatal assessment’ (*β *= 1.26, *p *< 0.001), ‘management of accidental dural puncture’ (*β *= 1.40, *p *< 0.001), and ‘confidence in providing anaesthesia for parturients with cardiovascular disease’ (*β *= 0.92, *p *< 0.001). In multivariate analysis, only two variables remained statistically significant as independent predictors: ‘confidence in providing anaesthesia for parturients with cardiovascular disease’ (*β *= 0.61, 95% CI: 0.217–0.997, *p *= 0.002) and ‘competence in managing neuraxial anaesthesia for caesarean section’ (*β *= 1.214, 95% CI: 0.190–2.238, *p *= 0.020).

**Table 4.** Regression of predictors for self-assessed confidence in obstetric anaesthesia training.

	Univariate			*p*-value		Multivariate			*p*-value	OR
	Beta	95% CI			Beta		95% CI			
Gender	0.92	(0.22,1.61)		0.01	−0.25	(0.24,2.50)		0.674	0.78
**Hospital level**									
Medical centre	−0.01	(−1.29,1.27)		0.989	−0.93	(0.40,4.17)		0.440	0.40
Regional hospital	ref	–		–	–	–		–	–
**Years of experience**									
Resident	−2.69	(v3.72,−1.67)		0.000	−1.70	(0.02,1.66)		0.131	0.18
≤5 years as attending	−0.92	(−1.80,−0.04)			−0.33	(0.11,4.62)		0.726	0.72
>5 years as attending	ref	–		–	–	–		–	–
**Training duration**									
Untrained	−22.4	(−6011,60068)		1.00	−13.4	00		0	0
1–3 months	−1.13	(−1.99,−0.27)		0.01	0.58	(0.38,8.35)		0.466	1.78
3–5 months	−0.93	(−1.81,−0.06)		0.04	0.60	(0.36,9.30)		0.470	1.82
>5 months	ref	–		–	–	–		–	–
Familiarity with physiological changes	1.51	(0.99,2.03)		0.000	0.70	(0.88,4.65)		0.098	2.02
Familiarity with the neonatal assessment	1.26	(0.81,1.72)		0.000	0.16	(0.55,2.49)		0.682	1.17
Management of accidental dural puncture	1.40	(0.92,1.88)		0.000	0.18	(0.51,2.78)		0.682	1.19
Management of maternal resuscitation	0.87	(0.55,1.18)		0.000	−0.02	(0.53,1.80)		0.942	0.98
Confidence in cardiac parturients	0.92	(0.60,1.24)		0.000	0.61	(1.24,2.71)		0.002	1.84
Familiarity with general anaesthetics	1.09	(0.66,1.52)		0.000	−0.19	(0.36,1.91)		0.654	0.83
Neuraxial anaesthesia for caesarean delivery	2.02	(1.34,2.70)		0.000	1.21	(1.21,9.38)		0.02	3.37

### Perspectives on undertaking additional electives

The qualitative analysis of open-ended responses from participants who were unwilling to allocate elective obstetric anaesthesia training identified several key considerations, summarised in Table 5. Some felt their current training was adequate due to frequent exposure during routine duties and on-call shifts, while others cited low case volume or repetitive content—particularly the emphasis on epidural placement—as limiting educational value. A subset expressed little interest in obstetric anaesthesia and preferred other subspecialties such as critical care, paediatric anaesthesia, or non-operating room anaesthesia. Additional concerns included poor interdisciplinary communication, perceived disrespect from obstetric staff, and anxiety about the specialty’s high-risk, unpredictable workload and medicolegal burden. Several respondents also noted that rare but critical emergencies may be better addressed through high-fidelity simulation than by extending rotation duration.

**Table 5.** Reasons for unwillingness to select additional obstetric training.

**Perceived Sufficiency of Current Training**
Training is already sufficient; obstetric cases are encountered during on-call shifts. The case volume at the training hospital is already adequate. I am already too exhausted. On-call shifts provide ample exposure to obstetric cases.
**Insufficient Obstetric Volume or Case Diversity**
The current training is too repetitive, focusing mainly on epidural procedures with limited exposure to other techniques. Additional months only reinforce technical aspects. Obstetric anaesthesia depends heavily on the hospital type. For low-delivery-volume hospitals, extending training is not helpful. Training for rare emergencies cannot be achieved merely by extending time; simulation-based training should be implemented regularly.
**Lack of Interest in Obstetric Anaesthesia**
I am more interested in other elective subspecialties. I prefer subspecialties such as non-OR anaesthesia or pain management. I lack interest in obstetric anaesthesia. My future workplace is unlikely to involve obstetric anaesthesia. With the declining birthrate, other areas of training are becoming more important.
**Issues in Training Structure and Institutional Readiness**
Elective months should address institutional weaknesses. Attending physicians are either unwilling or unable to provide training.
**Concerns Regarding Working Environment and Medico-Legal Risk**
I dislike the delivery room environment. Obstetricians often do not respect anesthesiologists. Obstetric anaesthesia is associated with high medicolegal risk;. The work is unpredictable and carries high risk.

## Discussion

To our knowledge, this is the first nationwide survey to systematically evaluate the current state and unmet needs of obstetric anaesthesia training in Taiwan. Despite the relatively low response rate, the structural alignment between our sample and the national clinician population suggests that our findings provide a representative snapshot of the Taiwanese obstetric anaesthesia community, particularly within high-volume medical centres where most residency training occurs. Whether reduced training exposure may contribute to increased maternal mortality is an important question; however, our data are insufficient to address this issue. Broader socioeconomic and demographic shifts, such as increasing maternal age, may also contribute. Although nationwide obstetric volumes have declined, simply extending rotation duration may not substantially increase clinical exposure. In low-volume centres, longer rotations do not necessarily increase case diversity, and rare emergencies cannot be reliably encountered by adding training time. Some trainees also perceived current exposure as adequate or repetitive and expressed greater educational needs in other subspecialties. Importantly, residents reported low confidence in managing critical obstetric anaesthesia emergencies. More broadly, the descriptive pattern in Table 2 suggests higher self-assessed competence in techniques commonly reinforced across anaesthesia practice, whereas several obstetric-specific or low-frequency situations, such as amniotic fluid embolism and neonatal resuscitation, received lower ratings. This pattern may reflect fewer opportunities for repeated clinical exposure to obstetric-specific emergencies. Cross-institutional partnerships may provide the high-acuity, supervised clinical experiences needed to develop competence in these rare but life-threatening scenarios. In Taiwan, some hospitals with limited paediatric or cardiac anaesthesia cases have used inter-institutional collaborations with larger medical centres to supplement trainees’ clinical exposure; a similar model could be considered for obstetric anaesthesia. Integrating competency-based education and entrustable professional activities into the curriculum may further allow high-stakes but infrequent tasks to be defined, observed, and evaluated despite limited clinical case availability.

Male respondents initially reported higher self-assessment ratings despite similar training exposure; however, male participants were also notably older. In multivariable regression, the association between gender and confidence disappeared after adjustment for professional rank and clinical seniority, suggesting that the observed difference primarily reflects an experience gap rather than an inherent gender effect. Nevertheless, gender-related workplace expectations may still influence the development of professional confidence. Similar findings have been reported in other specialties, where female residents reported lower confidence and higher burnout despite comparable training [23], were encouraged to ‘show more confidence’ rather than affirmed as leaders [24], and were more often expected to demonstrate politeness and compliance rather than initiative and leadership [25]. The ability to administer anaesthesia for high-risk obstetric patients and to perform neuraxial anaesthesia for caesarean delivery were the strongest predictors of overall self-confidence. This suggests that key technical skills and effective responses to high-pressure situations are central to the development of clinical confidence. Although theoretical knowledge and neonatal assessment were correlated with self-confidence, they did not demonstrate independent predictive value, suggesting that their contribution to overall confidence may be less direct. Ende et al. identified barriers discouraging residents from obstetric anaesthesia fellowships, including perceived lack of necessity, financial pressures, preference for other subspecialties, inadequate curriculum, limited enthusiasm, perceived benefit primarily for academic careers, and concerns regarding recognition status [26]. Conversely, over 90% of North American obstetric anesthesiologists reported career satisfaction, which was associated with meaningful work and collegial relationships [27]. In our study, negative training environments and interpersonal dynamics discouraged some trainees, underscoring the influence of workplace atmosphere on subspecialty interest. These findings suggest that decisions to pursue further obstetric anaesthesia training are shaped by educational, clinical, and cultural contexts. Simulation-based training may provide an important complementary strategy for addressing reduced clinical exposure, particularly for rare, high-acuity obstetric emergencies that cannot be reliably encountered during routine rotations.

High-fidelity manikin-based simulation enables repeated practice of crisis management, technical skills, and interprofessional teamwork in a controlled environment [28]. Virtual simulation may further broaden exposure without depending on actual case availability; a randomised controlled trial of immersive VR training in obstetrics demonstrated improvements in knowledge and confidence compared with conventional video-based learning [29]. AI-supported virtual simulation represents an emerging approach to adaptive case scenarios and individualised feedback, although its effectiveness in obstetric anaesthesia education remains to be established. These modalities should complement, rather than replace, supervised clinical experience. Repeated practice and structured feedback are also important, as specific procedural steps remained frequently overlooked eight months after simulation training for emergency caesarean delivery [30]. Finally, the declining birthrate has reduced obstetric case volumes and opportunities for clinical practice, while the efficiency- and cost-driven structure of Taiwan’s National Health Insurance system may further reduce physicians’ motivation to engage in obstetric anaesthesia. In high-pressure clinical settings, supervisors may restrict or take over procedures, further limiting residents’ hands-on experience. These structural and educational constraints highlight the importance of supportive learning environments that facilitate progressive skill acquisition and clinical judgement [31].

### Limitations

This study has several limitations. First, the 8.3% response rate subjects our findings to potential non-response and selection bias. Individuals with stronger interests in obstetric anaesthesia or those experiencing greater training challenges may have been more likely to participate, potentially skewing the results. Second, the observed gender differences in self-assessment are likely confounded by clinical seniority. Since male respondents were significantly older, this disparity may primarily reflect an "experience gap" rather than an inherent gender trait. Third, the reliance on self-reported data from a cross-sectional design may introduce subjective perception, social desirability, and recall bias, particularly among attending physicians reflecting on past training. Furthermore, the risk of Type I error due to multiple statistical comparisons must be acknowledged. Given the study's exploratory nature, results with high significance (*p *< 0.001) were prioritised for interpretation, though they should be viewed as preliminary trends.

## Conclusion

This study identifies critical gaps in Taiwan's obstetric anaesthesia training amid declining birthrates and shortened residency rotations. While clinical seniority correlates with higher perceived competence, urgent educational reforms—including high-fidelity simulation and inter-institutional partnerships—are necessary to address deficiencies in high-risk emergency management. Furthermore, fostering gender sensitivity and supporting early-career clinicians are vital to ensure equitable professional development and maintain maternal safety.

## Supplementary Material

Supplementary Material Supplementary material Obstetric Anesthesia Questionnaire English.docx

## Data Availability

The datasets generated and/or analysed during the current study are not publicly available due to ethical and privacy considerations for survey participants, but are available from the corresponding author upon reasonable request.
