# Supplementary material for: Evaluating perceived obstetric anesthesia training needs in residency education: a Taiwanese national survey
Source: Med Educ Online. 2026 Sep 15;31(1):2731792. doi: 10.1080/10872981.2026.2731792 (PMC13580366; doi:10.1080/10872981.2026.2731792)
Supplement: Supplementary file 1 [file ZMEO_A_2731792_SM4443.docx]

# Obstetric Anesthesia Training Questionnaire (English Version)

## Basic Information

1. What is your gender? (a) Male (b) Female

2. What is your age?

3. What is your training level? (a) PGY1 resident (b) PGY2 resident (c) PGY3 resident (d) PGY4 resident (e) Attending anesthesiologist ≤ 5 years (f) Attending anesthesiologist > 5 years

4. In which region is/was the hospital where you received obstetric anesthesia training located? (a) Northern Taiwan (b) Central Taiwan (c) Southern Taiwan (d) Eastern Taiwan

5. What is/was the level of the hospital where you received obstetric anesthesia training? (a) Medical center (b) Regional hospital

6. What is/was the total duration of your obstetric anesthesia rotation? (a) Not yet (b) 1–3 months (c) 3–5 months (d) More than 5 months

7. During your obstetric anesthesia rotation, on average, how many obstetric anesthesia cases did you encounter per month?

8. In your training institution, how many obstetric anesthesia experts were available to supervise your training?

## Knowledge and Skills in Obstetric Anesthesia

Please rate from 1 (very much needs improvement/learning) to 9 (no need for improvement/learning).

9. How familiar are you with the physiological changes during pregnancy?

10. How familiar are you with anesthetic drug selection for general anesthesia in obstetric patients?

11. How confident are you in managing the airway during general anesthesia in obstetric patients?

12. How familiar are you with the effects of general anesthetics on breastfeeding?

13. How familiar are you with the effects of general anesthetics on neonatal evaluation?

14. How confident are you in performing neuraxial anesthesia techniques for cesarean delivery (e.g., epidural, CSE, spinal)?

15. How familiar are you with the effects of local anesthetics in obstetric patients?

16. How familiar are you with the safety of local anesthetics in obstetric patients?

17. How familiar are you with the timing of withholding anticoagulants in obstetric patients?

18. How familiar are you with enhanced recovery protocols after cesarean delivery?

19. How confident are you in providing labor analgesia?

20. How confident are you in managing postoperative pain after cesarean delivery?

21. How confident are you in managing accidental dural puncture?

22. How confident are you in managing local anesthetic systemic toxicity?

23. How confident are you in managing high/total spinal block?

24. How confident are you in managing postpartum hemorrhage?

25. How confident are you in managing preeclampsia and eclampsia?

26. How confident are you in managing amniotic fluid embolism?

27. How confident are you in managing maternal cardiac arrest?

## Satisfaction and Confidence

Please rate from 1 (very dissatisfied / not confident / very unwilling) to 9 (very satisfied / very confident).

28. How confident are you in independently performing obstetric anesthesia (including GA, neuraxial anesthesia, postoperative pain management)?

29. How confident are you in providing anesthesia for pregnant patients with severe cardiovascular diseases (e.g., congenital heart disease, valvular disease, cardiomyopathy, heart failure, pulmonary hypertension)?

30. How satisfied are you with the number of hours spent in obstetric anesthesia rotation during your training?

31. How satisfied are you with the content of your obstetric anesthesia rotation during training?

32. Would you be willing to spend part of your elective months to increase obstetric anesthesia training? (a) Yes (b) No

33. If you are not willing, why not? (open-ended)

## Training Improvement

34. Which areas in obstetric anesthesia training do you think need to be strengthened? (multiple choice) (a) Knowledge (b) Technical skills (e.g., epidural placement) (c) Labor analgesia management (d) Management of maternal complications (e) Management of maternal emergencies

35. Please indicate any additional areas that need strengthening (open-ended).

36. Which teaching methods do you think could improve the effectiveness of obstetric anesthesia training? (multiple choice) (a) Real case observation (b) Simulation-based training (c) Online courses

37. Please indicate any additional teaching methods that could improve obstetric anesthesia training (open-ended).
